# Supplementary material for: Omega 3 Blends of Sunflower and Flaxseed Oil—Modeling Chemical Quality and Sensory Acceptability
Source: Foods. 2024 Nov 21;13(23):3722. doi: 10.3390/foods13233722 (PMC11640067; doi:10.3390/foods13233722)
Supplement: Supplementary file 1 [file foods-13-03722-s001.zip › foods-3250729-supplementary.pdf]

Supplementary material

Omega 3 Blends of Sunflower and Flaxseed Oil—Modeling Chemical Quality and Sensory Acceptability

Ranko Romanić [1,*], Tanja Lužaić [1], Lato Pezo [2], Bojana Radić [1,3] and Snežana Kravić [1]

[1] Faculty of Technology Novi Sad, University of Novi Sad, Bulevar cara Lazara 1, 21000 Novi Sad, Serbia
[2] Institute of General and Physical Chemistry, University of Belgrade, 11000 Belgrade, Serbia
[3] Institute of Food Technology in Novi Sad, University of Novi Sad, Bulevar cara Lazara 1, 21000 Novi Sad, Serbia
* Correspondence: rankor@uns.ac.rs; Tel.: +381-21-485-3700

Table S1. Elements of matrix W1 and vector B1 (presented in the bias column).

	1	2	3	4	5	6
S ratio	1.864	–0.014	1.925	3.889	1.837	0.479
F ratio	–2.966	–1.314	–3.266	–5.991	–2.786	–0.555
Bias	–1.054	–1.124	–1.422	–2.019	–0.975	–0.195

Table S2. Elements of matrix W_2 and vector B_2 (presented in the bias column).

Parameter	1	2	3	4	5	6	Bias
Color	–0.437	–0.966	0.000	–0.548	2.047	–0.675	0.875
Odor	–0.431	1.025	0.296	0.589	–1.786	0.634	0.380
Taste	0.494	–0.892	–0.009	–0.380	1.258	–0.497	0.329
Average rating	–0.057	–1.015	0.131	–0.461	1.688	–0.378	0.319
Total acceptability	0.490	–0.769	–0.073	–0.439	1.358	–0.585	0.378
L*	0.797	0.136	0.337	0.008	–0.381	–0.926	0.663
a*	–0.546	0.040	0.551	0.320	–0.804	–0.410	1.133
b*	–1.364	0.074	0.013	–0.219	1.350	0.676	0.353
ΔE	–1.435	0.067	–0.044	–0.144	1.075	1.205	0.069
PV	0.183	0.428	–0.058	–0.123	0.422	0.403	–0.199
AnV	–0.407	0.771	–0.094	0.024	0.240	1.237	–0.556
TOTOX	–0.362	0.594	0.047	–0.030	0.371	0.976	–0.433
CD	–1.014	0.655	–0.408	–0.108	0.844	1.778	–0.795
CT	–1.357	1.362	–0.296	0.189	–0.058	2.630	–1.241
CD/CT	0.954	–2.264	0.472	–0.323	0.744	–2.972	2.336
AV	–0.056	–0.997	0.401	–0.087	0.312	–1.495	1.614
C16:0	–0.918	0.455	–0.091	0.072	0.357	1.942	–0.909
C16:1	–0.811	0.405	–0.185	0.136	–0.014	2.030	–1.024
C18:0	–0.585	–0.350	0.279	0.086	–0.037	–0.496	1.169
C18:1c	–0.070	0.430	0.208	–0.049	0.037	0.994	–0.452
C18:2n6	–0.357	0.747	0.056	–0.049	0.352	1.063	–0.465
C18:3n6	–0.046	0.061	0.507	0.096	–0.409	–1.562	1.692
C18:3n3	–0.505	–0.197	0.639	0.035	–0.006	–1.144	1.480
C20:0	–0.535	0.306	–0.059	0.070	0.193	1.507	–0.669
C20:1	–0.363	0.415	–0.227	–0.007	0.243	1.174	–0.601
C22:0	0.215	0.680	–0.056	–0.121	0.298	0.458	–0.210
C24:0	0.443	–1.415	–0.043	–0.414	1.425	–0.773	0.694

Table S3. The „goodness of fit" tests for the developed ANN model.

Parameter	χ^2	RMSE	MBE	MPE	r^2	Skew	Kurt	Mean	StDev	Var
Color	–0.004	0.075	0.009	1.670	0.942	–0.182	–1.061	0.009	0.078	0.006
Odor	–0.008	0.113	0.016	2.268	0.668	0.713	0.204	0.016	0.118	0.014
Taste	–0.012	0.134	–0.025	3.818	0.966	–0.092	–0.999	–0.025	0.138	0.019
Average rating	–0.001	0.046	–0.022	0.915	0.971	–1.272	2.269	–0.022	0.042	0.002
Total acceptability	–0.001	0.042	–0.006	1.011	0.981	–0.458	–0.477	–0.006	0.043	0.002
L*	–0.036	0.234	–0.056	0.651	0.825	–1.836	4.502	–0.056	0.239	0.057
a*	–0.009	0.117	0.027	6.250	0.983	0.113	0.090	0.027	0.120	0.014
b*	–0.129	0.447	–0.059	3.886	0.956	0.163	–1.219	–0.059	0.465	0.216
ΔE	–0.154	0.487	–0.223	6.520	0.932	–0.155	–1.025	–0.223	0.454	0.206
PV	–0.004	0.080	0.004	7.546	0.951	0.222	–1.047	0.004	0.084	0.007
AnV	–0.484	0.864	0.262	8.972	0.965	–0.150	0.433	0.262	0.864	0.747
TOTOX	–0.793	1.107	0.507	18.779	0.964	–0.582	–0.607	0.507	1.032	1.066
CD	–0.048	0.272	0.086	7.087	0.900	0.338	0.729	0.086	0.270	0.073
CT	–0.002	0.062	0.005	17.758	0.854	0.042	–1.818	0.005	0.065	0.004
CD/CT	–2.204	1.846	–0.003	16.306	0.688	–1.032	1.658	–0.003	1.936	3.747
AV	–0.008	0.114	–0.007	14.282	0.838	–0.432	–0.523	–0.007	0.119	0.014
C16:0	–0.009	0.116	–0.001	1.586	0.896	1.311	0.661	–0.001	0.122	0.015
C16:1	0.000	0.012	0.000	14.389	0.534	1.108	1.073	0.000	0.013	0.000
C18:0	–0.005	0.092	–0.003	1.966	0.960	–0.451	0.330	–0.003	0.096	0.009
C18:1c	–0.545	0.918	0.489	2.948	0.966	0.375	0.000	0.489	0.815	0.664
C18:2n6	–2.239	1.860	–1.141	5.137	0.989	–0.544	0.268	–1.141	1.541	2.376
C18:3n6	0.000	0.023	–0.009	1.718	0.857	–1.548	1.966	–0.009	0.022	0.000
C18:3n3	–2.527	1.976	–0.217	207.782	0.989	1.133	1.262	–0.217	2.060	4.244
C20:0	0.000	0.008	–0.001	4.588	0.912	0.400	–0.210	–0.001	0.008	0.000
C20:1	0.000	0.008	–0.001	5.299	0.598	1.774	4.356	–0.001	0.008	0.000
C22:0	–0.001	0.036	0.016	9.774	0.959	0.124	–0.769	0.016	0.034	0.001
C24:0	0.000	0.015	0.005	7.596	0.856	–0.656	–0.661	0.005	0.015	0.000

χ^2—reduced chi–square; RMSE—root mean square error; MBE—mean bias error; MPE—mean percentage error; r^2—coefficient of determination; Skew—skeweness; Kurt—kurtosis; Mean—mean of residuals; StDev—standard deviation of residuals; Var—variation of residuals.

Table S4. Values of the control parameters used as limiting factors in the optimization.

	Color	Odor	Taste	Average rating	Total acceptability
Score	≥ 2.8	≥ 2.8	≥ 2.8	≥ 2.8	≥ 2.8

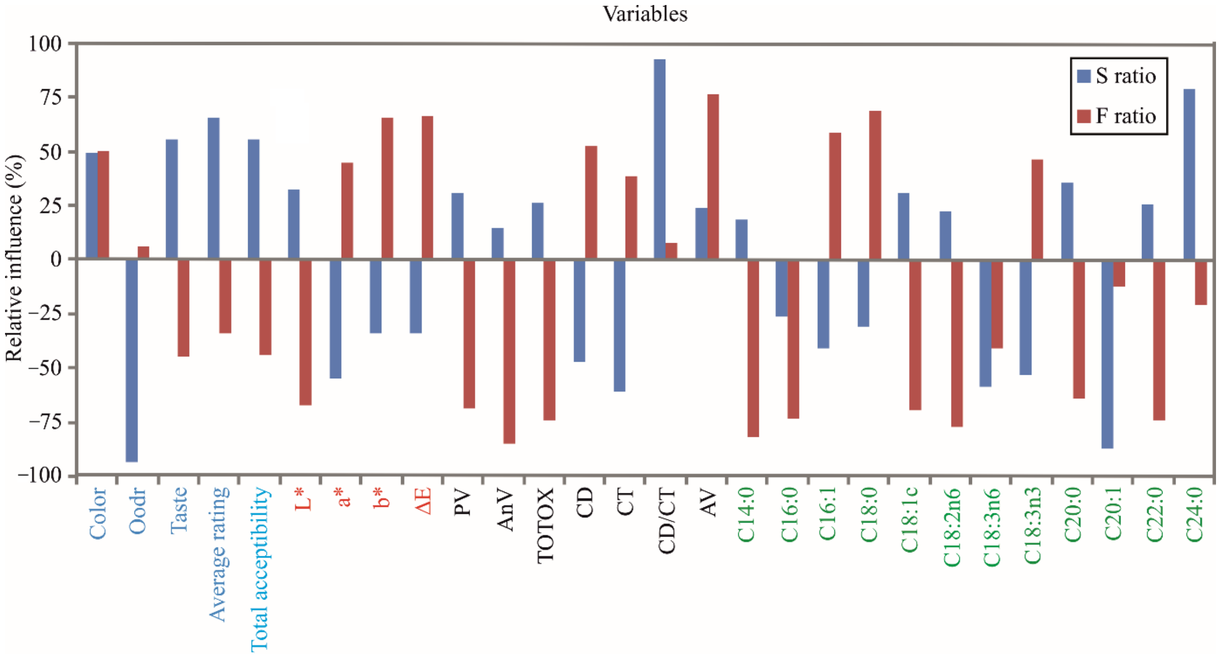

Figure S1. The influence of S (sunflower oil) and F (flaxseed oil) ratio input variables, identified on quality of oil blend regarding sensory analysis parameters (color, odor, taste, average rating, total acceptability), fatty acids content, oil quality (acid value—AV, peroxide value—PV, anisidine value—AnV, total oxidation value—TOTOX, conjugated dienes content—CD, conjugated trienes content—CT, conjugated dienes conjugated trienes ratio—CD/CT) and color characteristics (lightness—L*, redness—a*, yellowness—b*, total color difference—ΔE).
